# The efficacy of modified binding technique for renorrhaphy during robotic partial nephrectomy: surgical and functional outcomes from single-center experience

**DOI:** 10.1007/s00464-022-09460-y

**Published:** 2022-08-18

**Authors:** Zhi-Yu Wang, Wei Zhang, Shuan-Bao Yu, Yong-Hao Zhan, Ya-Feng Fan, Xue-Pei Zhang

**Affiliations:** 1 Department of Urology, The First Affiliated Hospital, Zhengzhou University, 1 Jian-She Road, Zhengzhou, 450052 Henan China; 2 Department of Pharmacy, Zhengzhou Orthopaedics Hospital, Zhengzhou, Henan China

**Keywords:** Renal cell carcinoma, Renal function, RENAL score, Renorrhaphy, Robotic partial nephrectomy

## Abstract

**Background:**

To compare the traditional single-layer and double-layer suture renorrhaphy with modified “Binding” suture renorrhaphy (whole rim of the wound was closed by the all-layer flow suture starting from the parenchyma cut edges to hilum, followed by the final defect closure) in robotic partial nephrectomy (RPN) for treating localized renal cell carcinoma in our large institutional experience.

**Methods:**

We retrospectively reviewed clinical data of 406 consecutive patients who underwent RPN from May 2018 and December 2020 in our center. The demographic and oncologic outcome variables were compared between different renal reconstruction groups and the effect of these suture techniques on renal function outcomes was also evaluated.

**Results:**

For the single-layer group, median operative time and warm ischemic time were significantly less than that of the double-layer and “Binding” groups (*p* < 0.001), while the significantly lower eGFR drop (*p* = 0.014) was also detected within postoperative 3 months from baseline, but this difference lost its statistical significance from 3th month to the last follow-up. The changes in postoperative creatinine values were clinically insignificant among the three groups. In a sub-analysis over 258 patients with moderate/high nephrometry score, those patients who underwent “Binding” suture had an undifferentiated warm ischemic time, estimated blood loss, and length of hospitalization stay with a decreased risk of Grade III complications (postoperative hemorrhage requiring intervention) and improved renal function recovery during the whole follow-up.

**Conclusion:**

Single-layer suture renorrhaphy may be associated with better renal functional preservation and could prove to be reliable in patients with low-complexity tumor (RENAL score ≤ 6). Patients with moderate/high-complexity tumor (RENAL score ≥ 7) might represent a subgroup of patients having a functional benefit after “Binding” suture renorrhaphy even in the long-term period.

**Graphical abstract:**

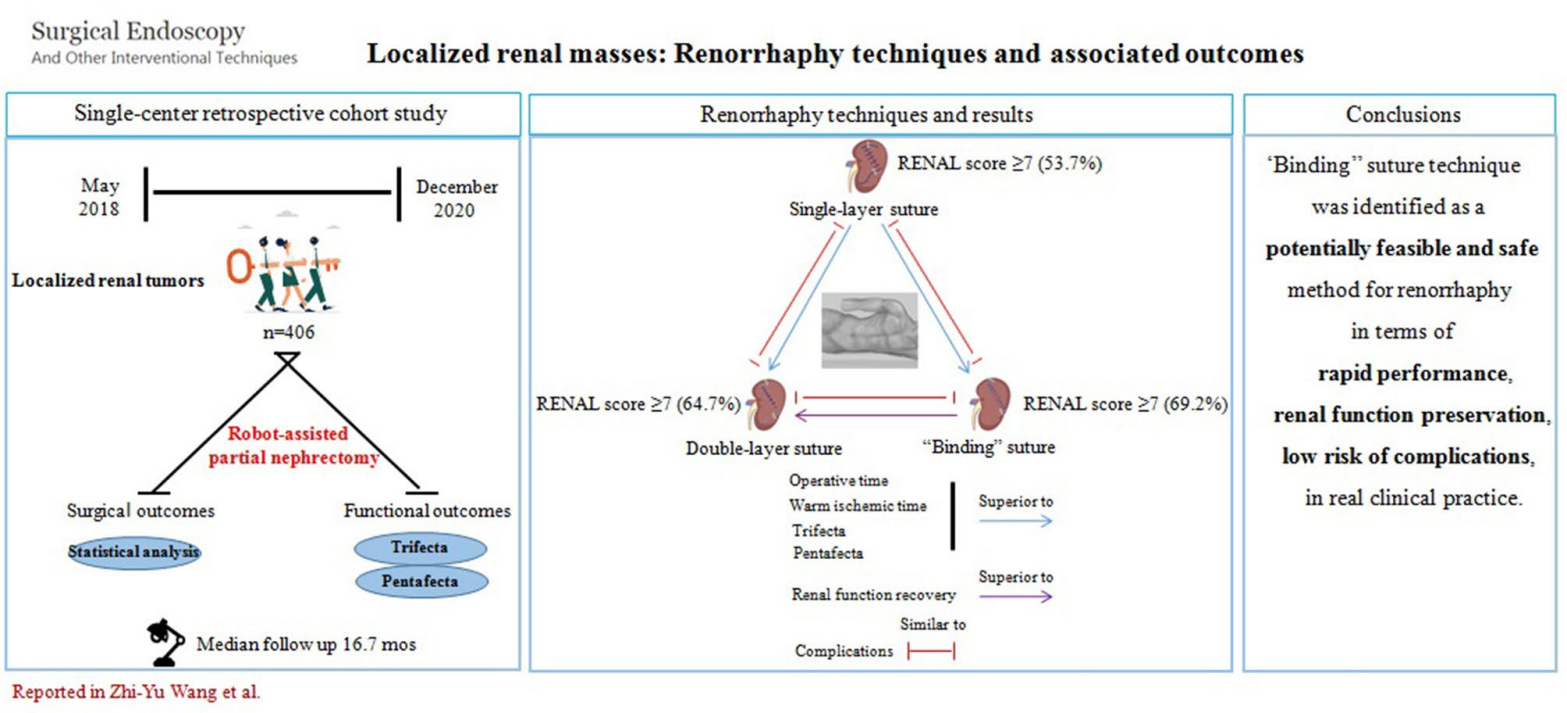

**Supplementary Information:**

The online version contains supplementary material available at 10.1007/s00464-022-09460-y.

Partial nephrectomy (PN) represents the standard treatment for localized renal tumors under the current guidelines [1, 2]. Even in cases of complex renal masses [3], PNs could be nowadays performed with minimally invasive techniques using robotic assistance [4, 5]. Noteworthy, the long-term implications of renal functional losses as a result of PNs have been increasingly recognized [6, 7], and various strategies to minimize the incidence of postoperative functional impairment have been reported. In this surgical setting, volume of preserved parenchyma, ischemia time, and maximal functional preservation are the main factors that affect functional recovery [8].

Despite advantages of robot-assisted partial nephrectomy (RPN) has been becoming well established, renorrhaphy remains a challenge in surgery. Renorrhaphy techniques during PNs have changed over the years mirroring the evolution of surgical experience, technology, complexity of renal tumors, and literature evidences. The predominant focus of renorrhaphy in early laparoscopic partial nephrectomy series (LPN) was to avoid bleeding complications and urinary leaks. Nowadays, renorrhaphy is also considered an essential determinant on long-term renal function [9]. To ensure a convenient and safe procedure, many renorrhaphy techniques have been devised and applied in clinical settings [10, 11]. However, renal reconstruction is differently performed and as a result there are no optimal reconstruction techniques recommended by current guidelines.

Previously, we had devised the “Binding” technique and showed its practicability for renorrhaphy by early clinical evaluation. In this paper, we attempted to compare the management difference of single-layer, double-layer, and “Binding” running techniques and evaluate the perioperative and postoperative oncologic and surgical outcomes in patients undergoing RPN. Besides, we carried out the first pooled literature analysis of the impact of these techniques on perioperative renal function change after RPN, and functional outcome (creatinine level and eGFR) during longer follow-up periods. The secondary aim of the study was to verify the clinical feasibility and safety of “Binding” technique in patients with moderate/high tumor complexity elements.

## Materials and methods

### Patient selection and surgical indication

From May 2018 and December 2020, the electronic medical records and surgical videos from 406 consecutive patients who underwent RPN for small renal masses were prospectively collected in our single center. Preoperative Computed Tomography (CT) or Magnetic Resonance Imaging (MRI) was used to obtain tumor parameters, including tumor location, tumor size, and tumor complexity according to RENAL nephrometry score [12]. In all cases, surgical access was achieved either transperitoneally or retroperitoneally. For the present series, a 4S Da Vinci robot (Intuitive Surgical, Sunnyvale, CA, USA) in a three-arm configuration was always used. The severity of postoperative complications was assessed according to the modified Clavien classification [13]. Patients were followed in outpatient department every 3 months during the first 2 years and every 6 months thereafter with thorax and abdomen CT scan. Renal function was assessed using the patient’s creatinine level and estimated glomerular filtration rate (eGFR) and by performing a renal scan. ^99m^Tc-DTPA renal scan was used for assessing eGFR in these patients. This study was approved by the local ethics committee, and individual informed consent was collected for all patients.

### Surgical technique

#### From patient positioning to port placement via transperitoneal approach

**Fig. 1:**
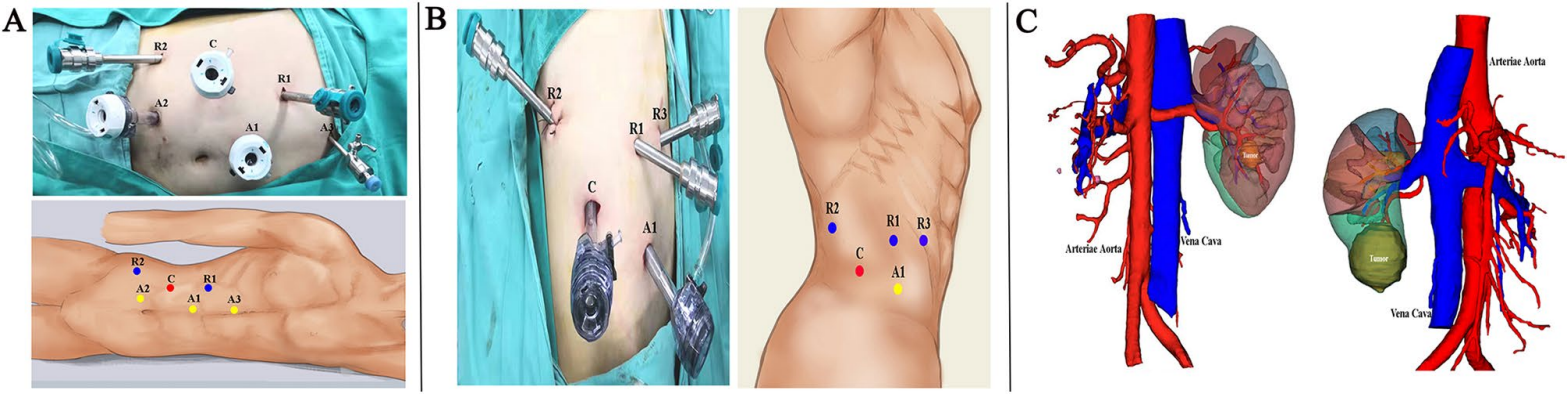
Patient position, port placement, and preoperative three-dimensional (3D) imaging. **A** Via transperitoneal approach (right side): C, trocar for camera; R1, robotic trocar for 1st arm; R2, robotic trocar for 2nd arm; A1, 12-mm trocar for assistant; A2, 12-mm trocar for assistant; A3, 5-mm trocar for assistant. **B** Via retroperitoneal approach (right side): C, trocar for camera; R1, robotic trocar for 1st arm; R2, robotic trocar for 2nd arm; R3, robotic trocar for 3rd arm; A1, 12-mm trocar for assistant. **C** 3D reconstruction from CT imaging showing the kidney mass, segmental arteries of the kidney, and renal calyces

Patients were placed in a lateral decubitus position at approximately 60 degrees when a nasogastric tube and Foley catheter were inserted preoperatively. Pneumoperitoneum was created by the use of a Veress needle. Basically, three robotic arm approaches, consisting of a 12-mm camera (30 degree downward lens) port and two robotic working ports (8 mm), were commonly used with two additional trocars for assistance, consisting of a nearly triangle shape with a 12-mm trocar placed in the midline between the camera port and the cranial working port. 5-mm auxiliary trocar was inserted under the xiphoid process (right lesion). The placement of trocars and their locations in RPN are presented in Fig. 1A.

#### From patient positioning to port placement via retroperitoneal approach

Patients were placed in full flank decubitus position with the ipsilateral side up relative to the renal tumor. The 12-mm trocar was placed 1–2 cm above the iliac crest for the laparoscope. The two 8-mm working ports were placed at the intersection of the posterior axillary line and the middle line of the costal margin and iliac crest. A fourth 8-mm trocar can be placed medially, in line with the other working port. Last, the assistant 12-mm trocar is in the middle line of camera trocar and the anterior 8-mm trocar above the anterior superior spine. The trocar configuration for retroperitoneal approach is illustrated in Fig. 1B.

#### Step-by-step surgical renorrhaphy procedures

Location of renal mass was approximately identified on the basis of preoperative imaging studies. Three-dimensional kidney reconstructions (3D printing models) of each patient were used for surgical planning and intraoperative surgical guidance (Fig. 1C). The surgical techniques (simple enucleation, enucleation with narrow healthy margin excision) to remove the tumor was determined based on preoperative imaging examination and intraoperative assessment. Tumors with distinct border displayed by radiography or ultrasonic contrast were mostly enucleated for all cases regardless of renorrhaphy patterns. Highly complex renal tumors (RENAL score ≥ 10) were mostly enucleated. An intraoperative flexible ultrasound is necessary for entirely endophytic tumors. With all approaches, only the renal main artery was clamped in all cases. All patients underwent RPN performed by one surgeon highly experienced in open and laparoscopic kidney surgery.

##### Single-layer running technique

**Fig. 2:**
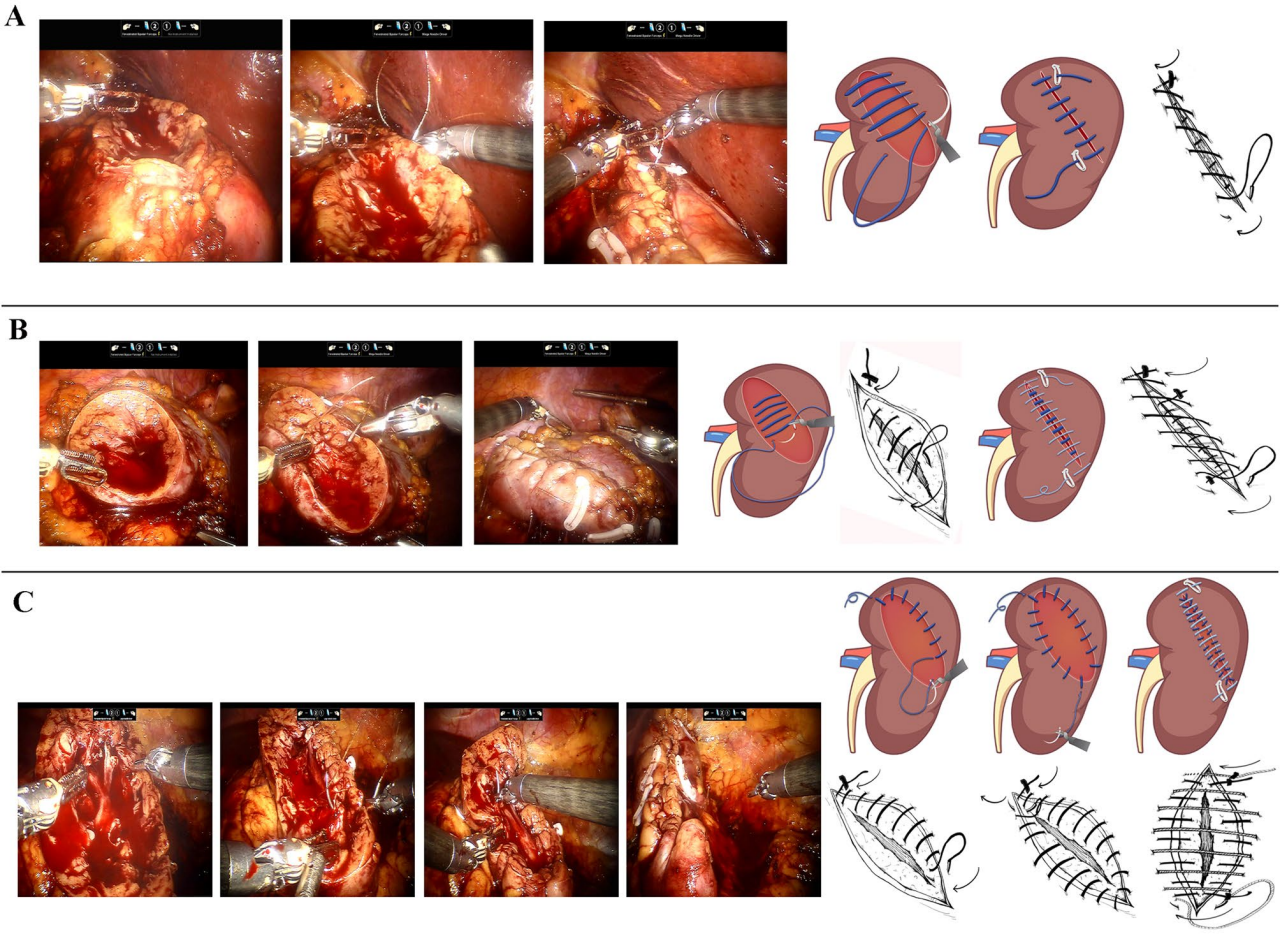
Intraoperative views and sketch map for brief description of renal reconstruction. **A** The parenchymal defect was closed with single-layer suture. **B** The parenchymal defect was closed with double-layer suture. **C** The parenchymal defect was closed with modified binding suture. Detailed line drawings showing the exact placement of running sutures in all 3 approaches displayed next to the relevant sketch map

Barbed thread (3–0 monofilament, 1/2 circle round-bodied needle) is passed through the outer renal capsule approximately 1.5 cm from the cut edge of the resection bed into the base of the renal defect in a running fashion, incorporating collecting system defects and blood vessels (Fig. 2A). Hem-o-Lok clips have been placed to anchor the initial and final throw outside the renal capsule. The surface of the kidney is covered by perirenal fat.

##### Double-layer running technique

Closure of the resection bed may be performed with aforementioned 3–0 monofilament-barbed suture in a running fashion, including repair of any vessel and collecting system defects. Sutures are anchored with a Hem-o-Lok clip. Outer layer of the renal capsule is closed using larger sutures and needles (0-QUILL) in a running fashion and secured with Hem-o-Lok clips (Fig. 2B). The surface of the kidney is covered by perirenal fat.

##### “Binding” running technique

After the tumor dissection is completed, the all-layer flow suture using 3–0 monofilament-barbed thread was applied starting from the parenchyma cut edges to hilum, first, and last suture is being fixed by clips on the renal capsule and finally the whole rim of the wound was closed. Afterward, a 0-QUILL suture is performed through the outer renal capsule approximately 1.5 cm from the cut edge of the resection bed into the base of the renal defect. A locking Hem-o-Lok is used to cinch the suture, compress the defect, and lock the suture in place (Fig. 2C). With the above key notes, most of nephron-sparing surgery can be successfully executed. Even for the hilar tumor, the cut parenchymal edge is achieved but rather radically coagulating the resection plane. In this circumstance, the absorbable hemostat is necessary in compressing the tiny defects.

### Statistical analysis

Data were performed as mean and standard deviation and median or percentage for continuous and categorical variables, respectively. The unpaired *t* test was used to compare continuous variables. The chi-squared test and Fischer exact test were used to compare categorical variables. All the data were analyzed by SPSS v.19.0 (IBM Corp. Armonk, NY, USA). Statistical significance was set at *P* < 0.05.

## Results

Patients, tumors characteristics, perioperative, and functional outcomes are summarized in Table 1. Mean age and body mass index were 53.4 years (range, 35–79 years) and 25.7 kg/m^2^ (range, 18.7–40.6 kg/m^2^), respectively. Clinical T1a cases represented 85.2%, 76.7%, and 73.7% of the single-layer, double-layer and “Binding” suture groups, respectively. The tumor characteristics expressed with the RENAL nephrometric scores indicated no differences between the three groups. Most percentage of tumors 296 (72.9%) showed an exophytic feature, with entirely endophytic 69 (17.0%) and cystic 41 (10.1%). The clear cell type was the majority of all cases 354 (87.2%), 10 (2.5%) papillary renal cell carcinoma, 3 (0.7%) chromophobe renal cell carcinoma, 20 (4.9%) Xp11.2 translocation renal cell carcinoma, and 19 (4.7%) benign.

**Table 1:** Comparison of demographics, perioperative outcomes, and follow-up characteristics from patients treated with single-layer, double-layer, and “Binding” suture techniques

Clinical parameters	SSR (*n* = 108)	DSR (*n* = 142)	BSR (*n* = 156)	*p value	**p value
Mean age (years)	51 (39–77)	56 (41–74)	52 (35–79)	0.005	
Gender (*n*, %)				0.023	
Male	60 (55.5)	99 (69.7)	110 (70.5)		
Female	48 (44.6)	43 (30.3)	46 (29.5)		
Body mass index (kg/m^2^)	25.7 (18.7–29.6)	24.3 (19.7–26.5)	31.2 (23.7–40.6)		
ECOG (*n*, %)				0.749	
0	89 (82.4)	122 (85.9)	132 (84.6)		
≥ 1	19 (17.6)	20 (14.1)	24 (15.4)		
Laterality (*n*, %)				0.805	
Left	45 (41.6)	64 (45.1)	75 (48.1)		
Right	60 (55.6)	75 (52.8)	79 (50.6)		
Bilateral	3 (2.8)	3 (2.1)	2 (1.4)		
RENAL score (*n*, %)				0.061	
6 or less	50 (46.2)	50 (35.2)	48 (30.8)		
7–9	40 (37.1)	53 (37.3)	62 (39.7)		
10 or greater	18 (16.7)	39 (27.5)	46 (29.5)		
Clinical T (*n*, %)				0.081	
T1a	92 (85.2)	109 (76.7)	115 (73.7)		
T1b	16 (14.8)	33 (23.3)	41 (26.3)		
Tumor location (*n*, %)				0.102	
Upper pole	27 (25.0)	51 (35.9)	55 (35.3)		
Mid pole	46 (42.6)	63 (44.4)	58 (37.2)		
Lower pole	35 (32.4)	28 (19.7)	43 (27.5)		
Tumor growth pattern (*n*, %)				0.024	
Exophytic	86 (79.6)	110 (77.4)	100 (64.1)		
Entirely endophytic	12 (11.1)	20 (14.1)	37 (23.7)		
Cystic	10 (9.3)	12 (8.5)	19 (12.2)		
Surgical path (*n*, %)				0.378	
Transperitoneal	78 (72.2)	102 (71.8)	122 (78.2)		
Retroperitoneal	30 (27.8)	40 (28.2)	34 (21.8)		
Operative time (min)	70.3 (55–98)	80.6 (77–108)	84.5 (68–110)	< 0.001	0.131
Warm ischemic time (min)	10.3 (9–20)	15.2 (14–24)	16.1 (12–28)	< 0.001	0.411
Estimated blood loss (ml)	100.3 (70–190)	116.5 (90–250)	127.8 (80–290)	0.109	0.920
Hospital stay (day)	5.2 (5–8)	5.8 (5–9)	6.1 (5–12)	0.091	0.331
Drainage	3.1 (2–6)	3.4 (3–7)	3.6 (3–7)	0.456	0.364
Renal function outcomes					
Serum creatinine (mg/dL)					
Preoperative	0.8 (0.6–2.6)	0.7 (0.7–2.8)	0.8 (0.6–3.2)	0.088	
Postoperative 24 h	1.0 (0.7–3.3)	0.9 (0.7–3.8)	1.0 (0.5–3.8)	0.068	0.251
Postoperative 1 month	0.9 (0.7–3.2)	0.9 (0.8–3.4)	0.9 (0.6–3.2)	0.101	1.000
Postoperative 3 months	0.9 (0.6–2.9)	0.8 (0.8–3.3)	0.9 (0.7–3.1)	0.094	0.988
eGFR (ml/min/1.73 m^2^)					
Preoperative	91.6 (85.5–113.5)	90.3 (86.0–116.5)	88.5 (85.2–110.2)	0.081	0.263
Postoperative 1 month	87.4 (76.6–100.3)	83.5 (72.4–98.6)	81.4 (69.8–91.8)	0.001	0.143
Postoperative 3 months	88.5 (79.6–102.7)	84.6 (75.4–101.1)	83.1 (76.4–96.8)	0.014	0.273
Grade and complications (*n*, %)				0.995	
No complication	87 (80.6)	112 (78.9)	126 (80.8)		
Grade I and Grade II	16 (14.8)	19 (13.4)	25 (16.0)		0.520
Grade III	5 (4.6)	11 (7.7)	5 (3.2)		0.082
Gross hematuria (*n*, %)				0.925	0.858
No	102 (94.4)	135 (95.1)	149 (95.5)		
Yes	6 (5.6)	7 (4.9)	7 (4.5)		
Perirenal hematoma (*n*, %)				0.715	0.488
No	101 (93.5)	132 (93.0)	148 (94.9)		
Yes	7 (6.5)	10 (7.0)	8 (5.1)		
Postoperative fever (*n*, %)				0.831	0.805
No	99 (91.7)	133 (93.7)	145 (92.9)		
Yes	9 (8.3)	9 (6.3)	11 (7.1)		
Embolization (*n*, %)				0.594	0.082
No	103 (95.4)	131 (92.3)	151 (96.8)		
Yes	5 (4.6)	11 (7.7)	5 (3.2)		

*BSR* “Binding” suture renorrhaphy, *DSR* double-layer suture renorrhaphy, *SSR* single-layer suture renorrhaphy, *ECOG* Eastern Cooperative Oncology Group, *eGFR* estimated glomerular function rate

*P value was calculated between three groups; **P value was calculated between double-layer suture and “Binding” suture groups

Most patients (74.4%) in three groups underwent transperitoneal RPN. Mean operative time was 70.3, 80.6, and 84.5 min in the single-layer, double-layer, and “Binding” suture groups, respectively (*p* < 0.001). Warm ischemic time (WIT) of single-layer group was significantly lower than the double-layer and “Binding” running groups (10.3 vs 15.2 min, 10.3 vs 16.1 min, *p* < 0.001). No statistically significant differences in aforementioned intraoperative variables were found between the double-layer and “Binding” running group (*p* = 0.131, *p* = 0.411, respectively). The estimated blood loss (EBL) and median postoperative hospital stay length were comparable among the three groups (*p* = 0.109, *p* = 0.091, respectively).

The groups were similar with regard to total incidence of medical and surgical complications (*p* = 0.995). Twenty patients (4.9%) were found postoperatively with hematuria (Clavien grade I), with no intervention taken. Twenty-five patients (6.1%) required intraoperative transfusions due to major hemorrhage or perirenal hematoma (Clavien grade II). Twenty-one patients (5.1%) received branch embolization after surgery, because of postoperative hemorrhage (Clavien grade III). Grade III complications were observed in 5 (4.6%) single-layer suture patients, 11 (7.7%) double-layer suture patients, and 5 (3.2%) “Binding” suture patients, respectively (*p* = 0.082). Although the tumors were relatively complex with respect to RENAL scoring (nephrometry scores ≥ 7 in 69.2% patients) in “Binding” suture group, the overall complication rate was slightly lower compared to the other two techniques (19.2%). No patient received open surgery. There were no grade IV and V complications, and there were no patients with delayed hemorrhage and urinary leakage, as of the latest follow-up in all patients.

**Fig. 3:**
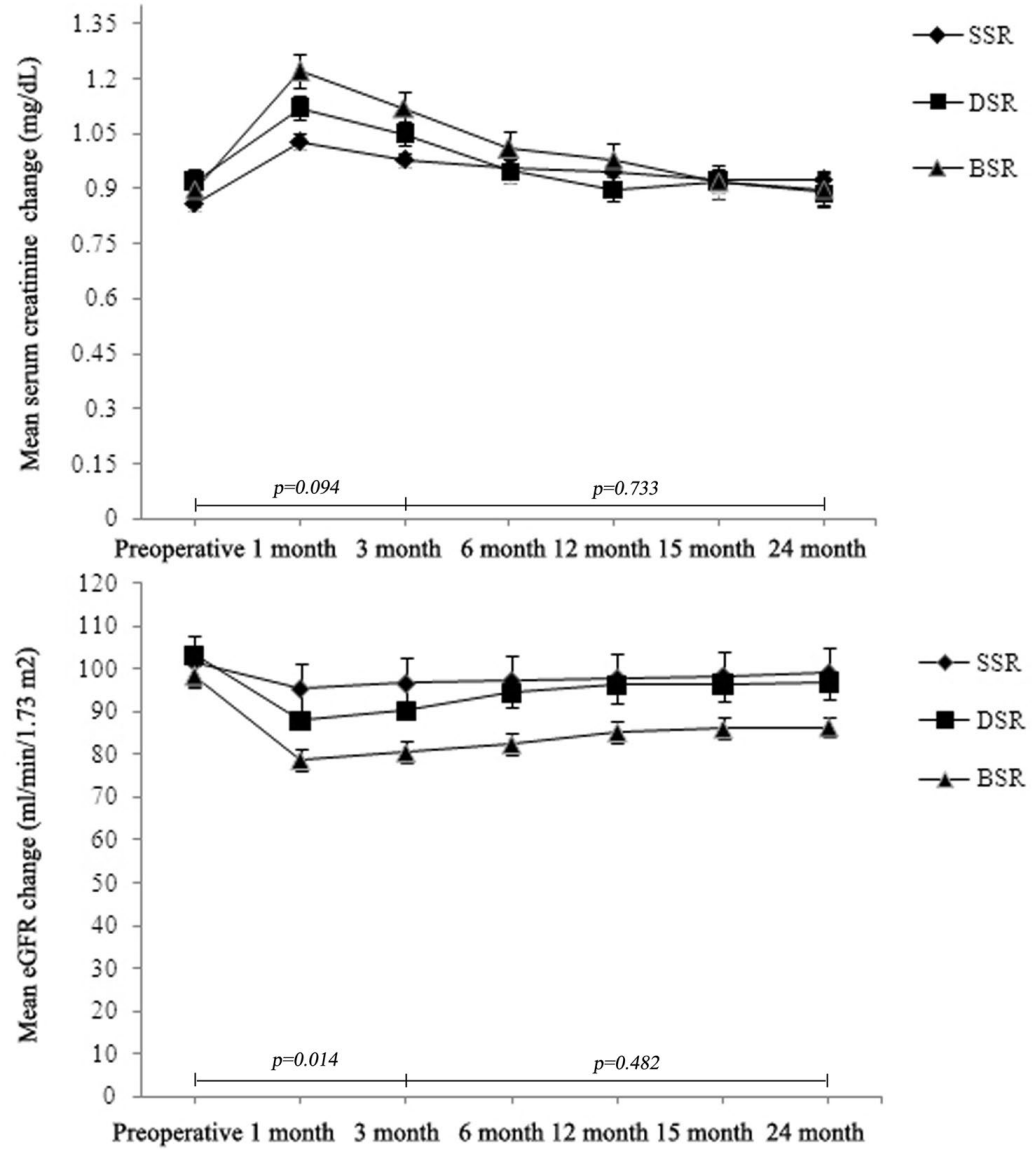
Effect of different renal reconstruction techniques on postoperative eGFR and serum creatinine over time. *SSR* single-layer suture renorrhaphy, *DSR* double-layer suture renorrhaphy, *BSR* “Binding” suture renorrhaphy

With a median follow-up period of 14.3, 18.3, and 17.6 months in three groups, respectively, patients submitted to “Binding” suturing had a higher median percentage of eGFR drop from baseline at first month (81.4 vs 87.4 ml/min/1.73 m^2^, *p* = 0.039) and at 3trd month postoperatively (83.1 vs 88.5 ml/min/1.73 m^2^, *p* = 0.047) compared to single-layer surgery technique; however, no detectable differences were observed in comparison with double-layer surgery technique (*p* = 0.143, *p* = 0.273, respectively). According to the preoperative results, the decrease in eGFR was commonly detected in the early postoperative period in this study, while long-term results were statistically similar after 3 months postoperatively, these results of which were in line with other study [14]. The trends of the absolute levels of eGFR during follow-up from baseline in each group are shown in Fig. 3. Of note, the mean drop in eGFR was marginally higher in the double-layer group and “Binding” group, which may partly be explained by relatively higher mean nephrometry scores. There was no statistically significant perioperative change in serum creatinine postoperatively (*p* = 0.068) (Fig. 3). Three double-layer surgery patients had local recurrence detected 8 months after surgery and underwent radical nephrectomy, and no recurrence was detected to date. Two patients had distant metastasis with no sign of local recurrence.

Taking into account these identifiable or unidentified selection biases (tumor complexity, surgeon’s preference and skills, perioperative eGFR, etc.), we further report on perioperative outcomes of the present RPN series with three suture techniques using Trifecta of outcomes previously defined [15]. Meanwhile, the Pentafecta criteria were also addressed. According to the new system, the goal of PN is reached when (1) surgical margins are negative, (2) WIT is ≤ 25 min, and (3) no perioperative complications are observed. And assessment of Pentafecta (the > 90% preservation of renal function and no stage upgrading of CKD (Chronic kidney disease) at 12 months postoperatively in addition to the Trifecta outcomes.

Functional, postoperative, and composite outcomes are described in Table 2. While performing the analysis, the rate of Trifecta for single-layer subgroup of our RPN cohort was 80.5% (87/108), double-layer 78.8% (112/142), and “Binding” 55.7% (87/156), respectively. In particular, no positive surgical margin was observed in the present RPN cases. The Pentafecta was achieved in 71 (65.7%), 69 (48.5%), and 85 (54.4%) of the patients in single-layer, double-layer, and “Binding” subgroups, respectively. Two hundred and twenty five (55.4%) of the patients preserved 90% of their preoperative eGFR. No patients were observed to upstage their CKD status to stage III–IV in the entire RPN cohort; however, 102 (25.1%) of patients upstaged their CKD status to stage II (data not shown).

**Table 2:** Postoperative functional outcomes

Variables	SSR (*n* = 108)	DSR (*n* = 142)	BSR (*n* = 156)	Total (*n* = 406)	*p value
No complication (*n*, %)	87 (80.5)	112 (78.9)	126 (80.8)	325 (80.0)	
Any Complications (*n*, %)	21 (19.4)	30 (21.1)	30 (19.2)	81 (19.9)	0.090
Positive Margin (*n*, %)	0	0	0	0	
WIT > 25 min (*n*, %)	0	0	39 (25.0)	39 (9.6)	< 0.001
Trifecta (*n*, %)	87 (80.5)	112 (78.8)	87 (55.7)	286 (70.4)	< 0.001
Pentafecta (*n*, %)	71 (65.7)	69 (48.5)	85 (54.4)	225 (55.4)	0.024
90% eGFR preservation (*n*, %)	71 (65.7)	69 (48.5)	85 (54.4)	225 (55.4)	0.024
Upstaged to CKD III–IV (*n*, %)	0	0	0	0	

*BSR* “Binding” suture renorrhaphy, *DSR* double-layer suture renorrhaphy, *SSR* single-layer suture renorrhaphy, *CKD* chronic kidney disease, *eGFR* estimated glomerular function rate, *WIT* warm ischemia time

*P value was calculated between three groups

## Discussion

Robotic surgery provides many potential advantages for renorrhaphy during PN, especially in case of complex or hilar masses [16–18]. Complete oncologic control as well as maximal functional preservation and avoidance of complications is the primary goal in RPN procedures. Current literature suggests that ischemia time and amount of healthy renal parenchyma resected are the main modifiable factors affecting renal function and volume loss after RPN [19]. Renal volume loss is thought to be more contributory to loss of renal function rather that ischemia time.

It is noteworthy that reconstruction techniques significantly affect the amount of vascularized parenchyma preserved, because a variable amount of healthy renal tissue is incorporated and potentially injured by renorrhaphy to achieve hemostasis, which leads to a major determinant of ultimate renal function [7, 20]. Renorrhaphy techniques have evolved over time due to several reasons, largely driven by surgical experience in addition to complexity of the tumor. Additionally, the approach taken for tumor removal can also have an impact on the type of reconstruction performed. Indeed, the achievement of cancer clearance after conservative renal surgery and the safety of the intervention are paramount.

It was previously reported that single-layer technique had better perioperative outcomes and a nonsignificantly higher rate of postoperative complications when compared with standard double-layer technique [21]. In this study, single-layer renorrhaphy could provide better several advantages than double-layer renorrhaphy in terms of mean operative time (70.3 vs. 80.6 min) and WIT (10.3 vs. 15.2 min) in our perioperative results. There were no differences in mean EBL (100.3 vs. 116.5 ml), duration of drainage (3.1 vs. 3.4 days), and length of stay (5.2 vs. 5.8 days) between the two groups. Additionally, perioperative eGFR was also compared between the two groups, with an eGFR loss of 4.6% for single-layer group vs. 8.0% for double-layer group at 1 month post-surgery, and it was clinically significant. On the one hand, permanent nephron damage may be caused by the more volume of perilesional healthy parenchyma excised in double-layer suturing group. In addition, the RENAL nephrometry score was ≥ 10 for 18 single-layer suturing cohort (16.7%) and 39 double-layer suturing patients (27.5%). Indeed, increasing RENAL nephrometry scores are correlated with longer WITs, sharper renal function decline, increased risk of intraoperative bleeding, and perioperative blood transfusion. However, the differences of postoperative overall complications rates and serum creatinine values in both groups were clinically insignificant during perioperative stage and subsequent follow-up (*p* = 0.743, *p* = 0.467).

From our entire study cohort, the overall operative time, the median WITs, and EBL were similar between double-layer suturing group and “Binding” suture group. Although the mean RENAL score was statistically insignificant between the two approaches and the HCT differences and hemoglobin were similar in both groups preoperatively, the rate of transfusion and re-intervention after bleeding was marginally higher in the double-layer group (3.2% vs 7.7%, *p* = 0.082). This may partly be explained by the pseudoaneurysm formation, which is a result of insufficient hemostasis within the resection bed [22]. As it is technically difficult during double-layer procedures to repair all the transected vessels with meticulous over-sewing or clipping. Besides in this study, two subgroups with clinical stage and RENAL score ≥ 10 were evaluated separately. The results showed that clinical T1b renal tumors and RENAL score ≥ 10 are associated with the risk of increased perioperative blood loss and transfusion, additionally in line with high rate of Clavien grade III (13/103, 12.6%). Although the median serum creatinine levels were slightly increased after RPN, there were no significant differences between the preoperative values and after 3 months postoperatively in both Groups.

The potential advantages of “Binding” suture renorrhaphy are as follows. First, the resolution in extensive wound closure is a challenging issue. We introduce the novel concept of “Binding” suture fashion, continuous barbed thread all-layer suturing was performed starting from the parenchyma to hilum, and finally binding shape of the closed wound was formed. Then, running stitches by 0-QUILL to tightly reapproximate the renal parenchymal edges around to cinch the defect closed. The absorbable hemostat gauze was optional for compression of the tiny venous tributaries bleeding. By applying the “Binding” suture technique, we have successfully performed RPN for more complex lesions with anatomic variation with mean RENAL nephrometry score of 7.4 and median tumor size of 5.5 cm.

Second, the core issues of the PN for hilar tumors are the fashions of dissection of tumors and suture of renal defects. A renorrhaphy technique which is effective for hemostasis but does not place undue tension on the branch vessels of the renal sinus remains one of the challenging steps after hilar tumor resection during RPN. The published V-hilar suture (VHS) technique is one option for reconstruction after an RPN involving the hilum [23, 24]. Our “Binding” technique can apply superior tension without injury and allow tighter renal parenchymal suturing without dehiscence. Due to the limited working space of suturing the parenchymal defect after hilar tumor excision, the all-layer flow suture surrounding the defect rims were introduced to reinforce renorrhaphy in order to minimize the resection bed space without deep layer suturing. In addition, hemostat gauze binding and suturing provide a double-wall blockage for bleeding on each side of the resection bed. Robotic partial nephrectomy with the “Binding” suture technique is a good alternative to VHS renorrhaphy in the management of renal hilar tumors.

Third, “Binding” suture renorrhaphy could be rapidly conducted with shortened clamping and suture time, as evidenced by the fact that mean operative time was 84.5 min and mean WIT was 16.1 min, making closing the parenchyma defect easier during the partial nephrectomy, which is superior to other studies [25, 26]. Moreover, the rate of overall complications was similar in single-layer and “Binding” groups. There was no significant decrease in renal function as measured by serum creatinine level and eGFR in this study compared to single-layer cohort after 3 months postoperatively. We hypothesized that the use of preoperative 3D print models maybe beneficial for understanding the vascular area and to calculate the tumor territories accurately, as a determinant of minimal suturing of the edge of the renal arterial branch and soft coagulation, which is important to preserve renal function during PN.

The study design was a case series with a small volume and was retrospective in nature, meaning some degree of bias was unavoidable. Our more strict definition described before as “Trifecta and Pentafecta” might be a better tool for assessing perioperative and functional outcomes after partial nephrectomy. The relatively higher mean rates of Trifecta achievement for the entire cohort were related to the significantly low overall percentage of positive surgical margin. The potential factors that may influence this outcome conclude: the modality of surgical approach (partial nephrectomy or radical nephrectomy), preoperative combined imaging analysis, variation in pathologist and pathology diagnostic techniques, and importantly, all cases were performed by single surgeon with less heterogeneity could also demonstrate lower positive margin rate to some extent. A significantly lower Trifecta rate was observed for “Binding” group compared to the other two groups, which may be explained by the extending warm ischemia time due to a relatively higher mean RENAL score in this group of patients. However, the rate of Trifecta for our entire RPN cohort was 70.4%, which is similar to the rate demonstrated by the recent literature [15]. Based on available evidence, in our single-center series single-layer subgroup was superior to the other two subgroups in terms of perioperative surgical outcomes measured by Trifecta and supplementary Pentafecta; there was no statistically significant difference between double layer and “Binding” cohort in terms of eGFR preservation and proportion of patients with CKD upstaging, but the superior rate of Pentafecta achievement for “Binding” suture predicted quick recovery for localized tumors.

Recently, unidirectional barbed suture material, performing a running or interrupted closure, has been applied to renorrhaphy in LPN and RPN and shown promising results in comparison with conventional polyglactin (Vicryl) running sutures [10, 27]. After each suture is tightened, the suture does not retract, avoiding the repeated replacement of Hem-o-Lok clip, which significantly shortens the operative and the ischemia time. After the clamp was removed, the evenly secure tightness of renal parenchymal suturing reduced the incidence of renal parenchymal tearing during renal reperfusion. Also in this study, barbed suture materials can be used for intracorporeal repair of renal parenchymal defects in RPN without slipping and fracture.

The results of our study should be carefully interpreted in view of several major limitations. First, surgeon experience and skills remain key factors for effective renal reconstruction, future studies with standardized reporting of resection and reconstruction techniques are needed to assess the real impact of such techniques on the early and long-term functional outcomes. Moreover, beyond the renorrhaphy technique, the materials used during renorrhaphy should ideally be compared to test their eventual impact. Furthermore, longer studies are necessary to allow a better assessment of perioperative complications between the various renorrhaphy techniques.

In a word, our study might be meaningful as an initial preliminary report demonstrating the impact of various renorrhaphy techniques on renal function following RPN. The potential advantages of “Binding” suture renorrhaphy may be mitigated as the intraoperative choice of the best renorrhaphy technique is almost always multifactorial.

## Supplementary Information

Below is the link to the electronic supplementary material.

Supplementary file1 (MP4 43957 KB)

Supplementary file2 (MP4 191187 KB)

Supplementary file3 (MP4 181939 KB)
